# Longitudinal renal function changes during real-world anti-vascular endothelial growth factor therapy for diabetic macular edema in Japan

**DOI:** 10.1007/s10384-025-01170-x

**Published:** 2025-02-28

**Authors:** Ayumi Usui-Ouchi, Shuta Kishishita, Yoshihito Sakanishi, Keitaro Mashimo, Kazunori Tamaki, Moe Matsuzawa, Meiko Kimura, Riyu Ikari, Shuu Morita, Ishin Ninomiya, Toshiro Sakuma, Nobuyuki Ebihara, Shintaro Nakao

**Affiliations:** 1 Department of Ophthalmology, Juntendo University Urayasu Hospital, 2-1-1 Tomioka, Urayasu, Chiba 279-0021 Japan; 2 Department of Ophthalmology, Juntendo University Graduate School of Medicine, 2-1-1 Hongo, Bunkyo-ku, Tokyo, 113-0033 Japan

**Keywords:** Diabetic macular edema, Anti-VEGF, Renal function, Diabetic retinopathy, Diabetic kidney disease

## Abstract

**Purpose:**

This retrospective observational study aimed to investigate the longitudinal changes in renal function and central macular thickness (CMT) and their impact on visual outcomes during anti-vascular endothelial growth factor (anti-VEGF) therapy for diabetic macular edema (DME).

**Study design:**

This study employed a retrospective observational design and analyzed data from treatment-naive patients with DME (62 cases, 100 eyes) receiving anti-VEGF therapy for 36 months. Baseline and follow-up assessments were conducted at 12, 24, and 36 months.

**Methods:**

Best corrected visual acuity (BCVA), CMT, number of anti-VEGF injections, HbA1c, serum creatinine (Cre), blood urea nitrogen (BUN), estimated glomerular filtration rate (eGFR), urinary protein levels, and chronic kidney disease (CKD) stage were measured at each time point.

**Results:**

The study population had a mean age of 60.7 ± 12.2 years, with 41 men and 21 women. Over the 36-month period, the mean number of anti-VEGF injections per eye was 5.3 ± 3.3. Maximum CMT significantly decreased at each time point, and final BCVA showed significant improvement (logMAR: − 0.07). HbA1c levels remained stable, BUN and Cre levels increased, and eGFR decreased significantly over time. CKD stage 3+ at 36 months significantly resulted in worse CMT.

**Conclusion:**

This retrospective observational study provides valuable insights into the longitudinal changes in renal function and CMT during anti-VEGF therapy for DME. Our findings emphasize the importance of monitoring renal function. This study contributes to our understanding of the complex relationship between renal function, DME, and anti-VEGF therapy, thereby facilitating improved management and outcomes in patients with DME.

## Introduction

Diabetic retinopathy (DR) is a common microvascular complication of diabetes and the leading cause of blindness in working-age adults worldwide. Approximately 28 million people are affected by vision-threatening diabetic retinopathies, including diabetic macular edema (DME) [1]. Globally, approximately 7% of individuals with diabetes are estimated to develop DME, and this number is expected to increase with the increasing prevalence of diabetes [1]. Currently, anti-vascular endothelial growth factor (anti-VEGF) drugs are the first choice for DME treatment; however, 30–40% of patients with DME exhibit poor responses to anti-VEGF drugs [2]. Diabetic kidney disease (DKD) is a manifestation of microvascular complications resulting from hyperglycemia in patients with diabetes. DR and DKD frequently coexist, representing a significant burden on affected individuals [3, 4]. Recently, DKD has been reported to be associated with the progression of DR and an increased risk of developing DME [3–6].

Additionally, retinopathy affects the progression of nephropathy [7, 8]. However, there is no consensus regarding the correlation between the degree of renal dysfunction and the responsiveness to anti-VEGF therapy for DME, because different studies report controversial results [9–12].

In this study, we retrospectively investigated longitudinal changes in renal function, central macular thickness (CMT), and best corrected visual acuity (BCVA) over more than 36 months in patients with DME undergoing anti-VEGF therapy. We examined the progression of renal impairment and the responsiveness to anti-VEGF therapy in real-world cases of DME.

## Material and methods

### Study design

We included consecutive treatment-naive patients with DME who received anti-VEGF therapy on a pro re nata (PRN) basis for more than 36 months and underwent annual blood tests between January 2015 and September 2022 at the Department of Ophthalmology, Juntendo University Urayasu Hospital. Patient data were collected retrospectively, and the following parameters were evaluated at baseline, as well as at 12, 24, and 36 months after the initiation of treatment: BCVA (logarithm of the minimum angle of resolution [logMAR]), CMT, number of anti-VEGF injections, HbA1c, blood urea nitrogen (BUN), serum creatinine (Cre), estimated glomerular filtration rate (eGFR), and presence of overt proteinuria. Patients with unclear OCT images or missing data at any time point within the 36-month period were excluded from the study. The CKD stages were categorized following the National Kidney Foundation guidelines: Stage 1: Kidney damage with normal or increased eGFR (≥90 mL/min/1.73 m^2^). Stage 2: Kidney damage with mildly decreased eGFR (60–89 mL/min/1.73 m^2^). Stage 3: Moderately decreased eGFR (30–59 mL/min/1.73 m^2^). Stage 4: Severely decreased eGFR (15–29 mL/min/1.73 m^2^). Stage 5: Kidney failure (GFR <15 mL/min/1.73 m^2^ or dialysis).

### Data analysis

All results are expressed as the mean and standard deviation (SD). Data were analyzed using the GraphPad Prism software (GraphPad). Longitudinal changes in the measured parameters were analyzed using appropriate statistical methods such as repeated-measures analysis of variance (ANOVA) or mixed-effects models. Post hoc analyses, including pairwise comparisons, were performed to assess specific time points or subgroups within the study population.The changes in BCVA and CMT before and after the course were analyzed using the Wilcoxon signed-rank test, and the changes in BCVA and CMT between the two groups were examined using the Mann-Whitney U test. Statistical significance was set at p<0.05.

### Ethical considerations

This study was conducted in accordance with the ethical principles outlined in the Declaration of Helsinki. Institutional Review Board approval was obtained from Juntendo Urayasu Hospital before data collection (approval number: 2020-065). Patient confidentiality and data protection were ensured throughout the study, and all data were de-identified and securely stored.

## Results

### Baseline characteristics

Baseline patient characteristics are presented in Table 1. A total of 62 cases, corresponding to 100 eyes, were included in this study. The mean age of the study population was 60.7 ± 12.2 years. Of the 62 patients, 41 were men, and 21 were women. The average duration of observation was 59.2 ± 15.2 months. At baseline, the mean logMAR BCVA was 0.53 ± 0.33. The mean CMT at baseline was 536.4 ± 161.4 μm. The average HbA1c level was 7.37 ± 1.17%. The baseline values for renal function parameters were: serum Cre = 1.02 ± 1.22 mg/dL, BUN = 18.0 ± 8.8 mg/dL, and eGFR = 76.0 ± 28.8 mL/min/1.73m^2^. A total of 26 cases (42%) tested positive for urinary protein. In the study population, 45 patients (73%) had CKD stage 2 or higher, whereas 12 (19%) had stage 3 or higher.

**Table 1:** Patients’ characteristics

Parameter	
Eyes/patients	100/62
Age, y	60.7 ± 12.2
Sex (male: female)	41 : 21 (66%: 36%)
LogMAR BCVA	0.53 ± 0.33
CMT, μm	536.4 ± 161.4
HbA1c (%)	7.4 ± 1.2
Systemic hypertension	31 patients
Serum creatinine, mg/dL	1.01 ± 1.22
Blood urea nitrogen, mg/dL	18.0 ± 8.8
eGFR (mL/min/1.73m^2^)	76.0 ± 28.8
Macroalbuminuria	26 patients (42%)
CKD G1	17 patients (27%)
CKD G2	33 patients (53%)
CKD G3	9 patients (15%)
CKD G4	1 patient (2%)
CKD G5	2 patients (3%)

*HbA1c* hemoglobin A1c, *LogMAR* logarithm of the minimum angle of resolution, *BCVA* best corrected visual acuity, *CMT* central macular thickness, *eGFR* estimated glomerular filtration rate, *CKD* chronic kidney disease.

### Teatment regimen and number of Anti-VEGF injections

The treatment regimen consisted of 63 eyes (63%) receiving three injections followed by PRN and 47 eyes (47%) receiving a single injection followed by PRN. Five eyes (5%) received monotherapy with ranibizumab, 23 eyes (23%) were switched from ranibizumab to aflibercept, and 72 eyes (72%) received monotherapy with aflibercept. Over the course of 3 years, the average number of anti-VEGF injections administered per eye was 5.3 injections (SD: 3.3 injections). Additionally, the average number of anti-VEGF injections administered per individual over the 3-year period was 8.5 injections (SD: 5.2 injections). The number of anti-VEGF injections decreased annually.

### Central macular thickness progression

The maximum and minimum CMT values for each year are listed in Table 2. In all yearly assessments, the minimum CMT was significantly lower than the maximum value (p<0.0001). The maximum CMT values in the third year were significantly lower than the maximum CMT observed in the first year (*p* = 0.0117).

**Table 2:** Course of changes in central macular thickness

Months	Number of injections (/eye)	Maximum CMT (μm)	Minimum CMT (μm)	*p-*value
Baseline–12	3.0 ± 1.5	536.5 ± 161.5	263.8 ± 91.6	*<0.0001
13–24	1.6 ± 1.7	505.8 ± 197.8	251.5 ± 90.5	*<0.0001
25–36	0.7 ± 1.2	449.3 ± 197.0**	244.1 ± 99.5	*<0.0001

*CMT* central macular thickness

*Comparison between maximum and minimum CMT using two-way ANOVA.

**Maximum CMT at each year was assessed compared to the first year by repeated measures ANOVA *p*<0.0001

### Visual acuity progression

Changes in BCVA were assessed by comparing BCVA at 36 months to baseline measurements. The BCVA at 36 months (0.45 ± 0.41) showed a significant improvement compared to baseline, with a change of − 0.07 logMAR over 3 years (*p* = 0.0318).

### Systemic factors change

**Fig. 1:**
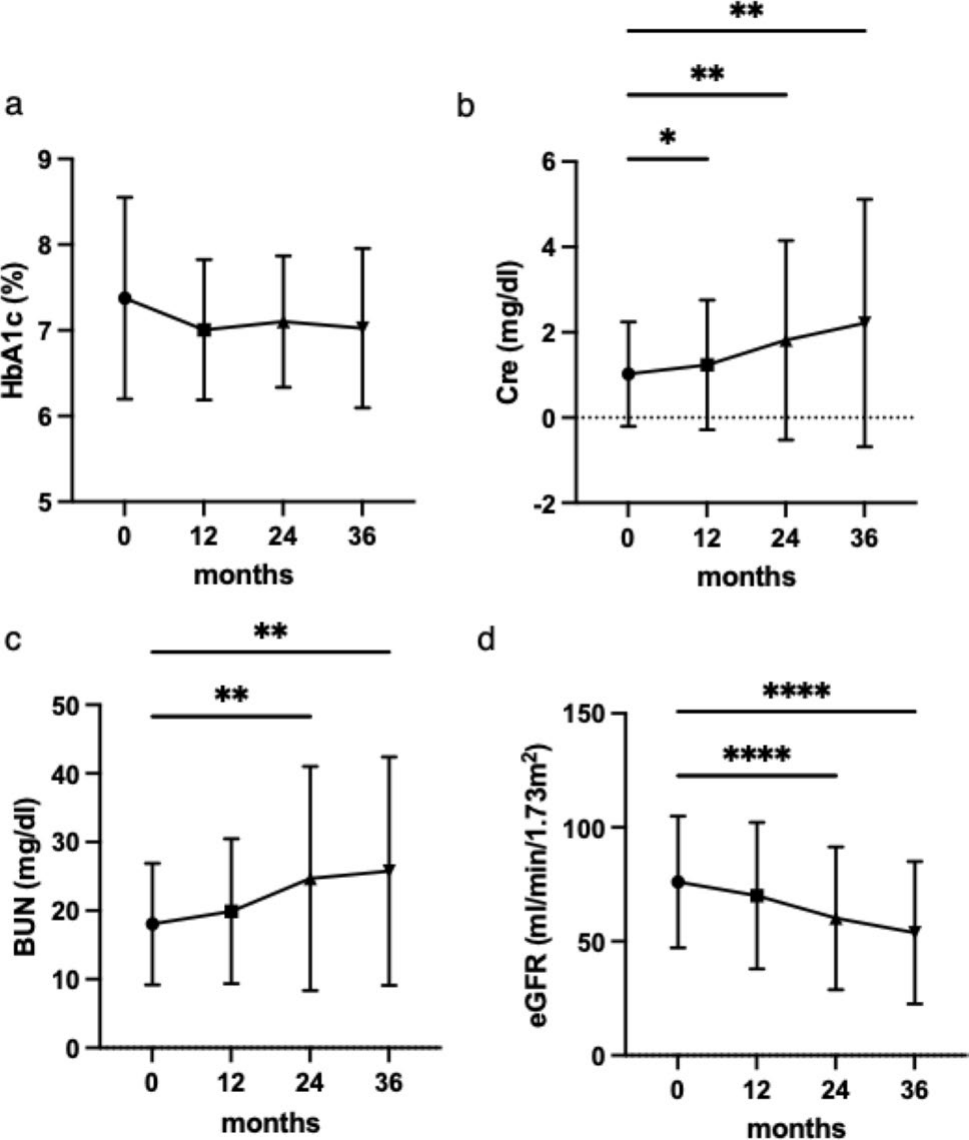
Longitudinal Changes in HbA1c, serum Cre, serum BUN, and eGFR. Longitudinal changes in HbA1c (**a**), serum creatinine (**b**), serum BUN (**c**), and eGFR (**d**) levels are shown. Statistical analysis was performed using repeated-measures ANOVA. *p<0.05, **p<0.01, ***p<0.001, and ****p<0.0001. *Cre* creatinine, *BUN* blood urea nitrogen, *eGFR* estimated glomerular filtration rate, *HbA1c* hemoglobin A1c, *Cre* creatinine, *BUN* blood urea nitrogen, *eGFR* estimated glomerular filtration rate

**Fig. 2:**
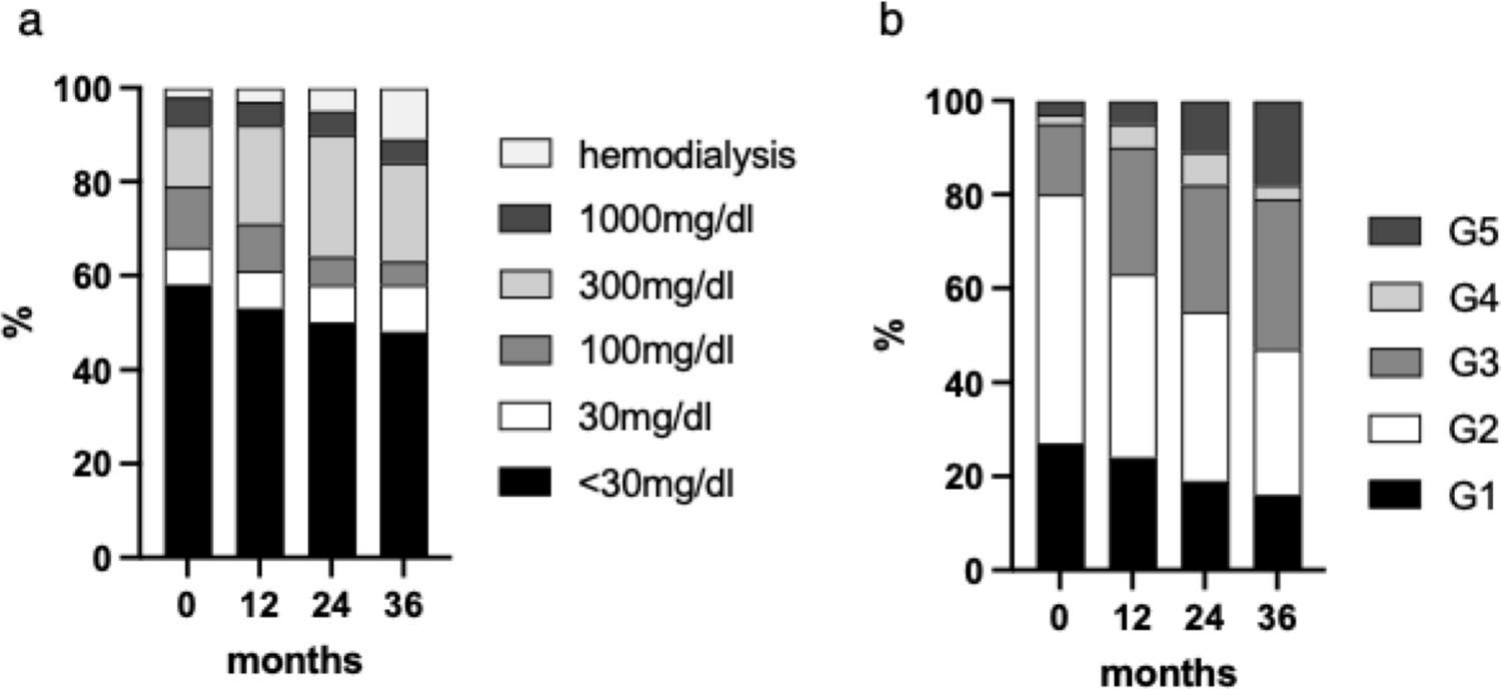
The longitudinal changes in urinary protein levels and CKD stages. Longitudinal changes in urinary protein levels (**a**) and CKD stage (**b**) are shown. *CKD* chronic kidney disease

While no significant changes in HbA1c were observed over time, all serum BUN, Cre, and eGFR levels showed a significant increase over the course of the study period compared to baseline (Fig. 1a–d). Changes in the urinary protein levels were also assessed over time. The presence of macroalbuminuria increased progressively throughout the study period (Fig. 2a). Additionally, we assessed changes in the CKD stage throughout the study period (Fig. 2b). At baseline, 72.6% of the study population had CKD stage 2 or higher, whereas 19.4% had CKD stage 3 or higher. After 36 months of treatment, the proportion of patients with stage 2 or higher CKD increased to 83.9%, and the proportion of patients with stage 3 or higher CKD increased to 53.2%.

When comparing the baseline characteristics, progression, and final outcomes based on the presence or absence of stage 3 CKD or higher (eGFR < 60) at baseline, no significant differences were observed in BCVA, CMT, or the number of anti-VEGF injections at any point (Table 3). This suggests that the presence of stage 3 CKD or higher at baseline did not significantly impact these outcomes. When examining the presence or absence of stage 3 CKD or higher (eGFR < 60) at 36 months, a significant difference was observed in the maximum CMT in the third year, showing that patients with stage 3 CKD or higher had a significantly higher maximum CMT than those without stage 3 CKD or higher although the minimum CMT was comparable (506.5 ± 201.4 vs. 404.6 ± 171.9, *p* = 0.011) (Table 4). The number of injections over the three-year period was equivalent between patients with stage 3 or higher CKD and those without stage 3 or higher CKD at 36 months (Table 4). These findings suggest that the presence of stage 3 CKD or higher at 36 months may be associated with greater CMT fluctuation, indicating potential implications for disease progression and treatment response.

**Table 3:** Course of visual acuity, central macular thickness, and number of anti-VEGF injections according to CKD stages at baseline

	eGFR<59 mL/min/1.73m^2^ (*n *= 20)	eGFR>60 mL/min/1.73m^2^ (*n *= 80)	*p-*value
Baseline BCVA logMAR	0.46 ± 0.24	0.55 ± 0.35	NS
Baseline CMT	492.4 ± 123.4	547.5 ± 168.5	NS
Minimum CMT at the first year	260.3 ± 99.1	264.6 ± 90.3	NS
Maximum CMT at the second year	443.0 ± 230.0	521.5 ± 187.4	NS
Minimum CMT at the second year	222.3 ± 77.9	258.8 ± 92.4	NS
Maximum CMT at the third year	427.4 ± 214.1	456.0 ± 193.4	NS
Minimum CMT at the third year	217.5 ± 83.7	251.0 ± 102.6	NS
BCVA at 36 months logMAR	0.45 ± 0.42	0.43 ± 0.37	NS
Number of anti-VEGF injections	4.8 ± 3.0	5.5 ± 3.6	NS

*LogMAR* logarithm of the minimum angle of resolution, *BCVA* best corrected visual acuity, *CMT* central macular thickness, *eGFR* estimated glomerular filtration rate, *CKD* chronic kidney disease.

**Table 4:** Course of visual acuity, central macular thickness, and number of anti-VEGF injections according to CKD stages at 36 months

	eGFR<59 mL/min/1.73m^2^ (*n *= 20)	eGFR>60 mL/min/1.73m^2^ (*n *= 80)	*p-*value
Baseline BCVA logMAR	0.46 ± 0.24	0.55 ± 0.35	NS
Baseline CMT	492.4 ± 123.4	547.5 ± 168.5	NS
Minimum CMT at the first year	260.3 ± 99.1	264.6 ± 90.3	NS
Maximum CMT at the second year	443.0 ± 230.0	521.5 ± 187.4	NS
Minimum CMT at the second year	222.3 ± 77.9	258.8 ± 92.4	NS
Maximum CMT at the third year	427.4 ± 214.1	456.0 ± 193.4	NS
Minimum CMT at the third year	217.5 ± 83.7	251.0 ± 102.6	NS
BCVA at 36 months logMAR	0.45 ± 0.42	0.43 ± 0.37	NS
Number of anti-VEGF injections	4.8 ± 3.0	5.5 ± 3.6	NS

*LogMAR* logarithm of the minimum angle of resolution, *BCVA* best corrected visual acuity, *CMT* central macular thickness, *eGFR* estimated glomerular filtration rate, *CKD* chronic kidney disease.

### CMT progression in cases requiring dialysis

Overall, 8 cases (12.9%) required dialysis during the follow-up period. In these cases, both edema recurrence and a reduction in retinal thickness due to anti-VEGF therapy were observed before dialysis initiation. Table 5 lists the eight cases that progressed to dialysis, detailing the treatment regimens and the efficacy of anti-VEGF therapy from three years prior to dialysis initiation. All patients who required dialysis during the follow-up period showed a significant decrease in CMT after starting dialysis, with only one patient requiring additional anti-VEGF injections (Table 5). Anti-VEGF therapy (aflibercept in all eight cases) remained at least partially effective even as renal function deteriorated up to the point of dialysis initiation (Table 5).

**Table 5:** The treatment course, visual acuity, and CMT changes in cases that progressed to dialysis during follow-up

Months before or after the initiation of dialysys	eGFR (mL/min /1.73m^2^)	Serum creatinine, (mg/dL)	Number of injections (/eye)	LogMAR	Maximum CMT (μm)	Minimum CMT (μm)	*p-*value
36M–25M pre	66.3 ± 15.6	1.0 ± 0.3	3.1 ± 1.8	0.7 ± 0.1	528.0 ± 175.6**	234.4 ± 116.3	*<0.0001
24M pre–13M pre	51.2 ± 37.3	1.6 ± 1.1	1.8 ± 2.0	0.5 ± 0.2	493.7 ± 273.4**	239.0 ± 125.5	*<0.0001
12M pre– 1M pre	18.0 ± 11.6	3.5 ± 1.5	0.4 ± 0.7	0.6 ± 0.3	454.5 ± 209.1**	188.0 ± 68.5	*<0.0001
0M post–12M post	6.0 ± 1.0	7.8 ± 1.2	0.2 ± 0.5	0.2 ± 0.2	212.9 ± 161.8	161.8 ± 55.9	NS

*CMT* central macular thickness

*Comparison between maximum and minimum CMT using two-way ANOVA.

**Maximum CMT at each year was assessed compared to post dialysis by repeated measures ANOVA *p*<0.01

## Discussion

This study aimed to investigate longitudinal changes in renal function and CMT during anti-VEGF therapy for DME. Our findings contribute to current knowledge by providing insights into the relationship between renal function, DME, and treatment outcomes. First, we observed a mean total of 5.3 anti-VEGF injections per eye over the course of 3 years (first year: 3.0, second year: 1.6, third year: 0.7). Compared with other real-world studies, such as STREAT-DME in Japan [13] (4.3 injections over 2 years), LUMINOUS (conducted in over 43 countries, including regions in Asia, Europe, North America, Latin America, and Oceania) [14] (4.5 injections over 1 year), MERCURY in Japan [15] (4.9 injections over 2 years), and Talks SJ et al. in the UK [16] (12 injections over 3 years), our study demonstrates an injection frequency comparable to those observed in the studies conducted in Japan. This consistency in the treatment frequency reflects the standard practice of managing DME using anti-VEGF therapy in a real-world setting in Japan.

Regarding visual outcomes, our study shows a mean improvement of − 0.07 logMAR over 3 years. This improvement is consistent with the findings from STREAT-DME (change of − 0.09 logMAR/2 years), LUMINOUS (change of − 0.07 logMAR/1 year), and MERCURY (change of − 0.13 logMAR/2 year). Notably, each study had a different follow-up duration [14, 15]. Nonetheless, these findings collectively highlight the efficacy of anti-VEGF therapy in improving the visual acuity in patients with DME. Comparing our results with those of previous studies, we observed similar treatment responses in terms of injection frequency and visual outcomes.

In our study, we found that 72.6% of the patients at baseline and 83.9% at 36 months after anti-VEGF therapy had stage 2 or higher CKD. This is comparable to Busch et al., who report that 83% of patients with DME had stage 2 or higher CKD [17]. The high prevalence of CKD in patients with DME highlights the close association between renal function impairment and the development or worsening of DME. These findings emphasize the importance of monitoring renal function in patients with DME and underscore its potential role in predicting DME exacerbation. The mechanism by which CKD exacerbates DME includes decreased plasma colloid osmotic pressure due to urinary protein excretion, elevated circulating levels of VEGF and inflammatory cytokines, disruption of the blood-retinal barrier, and increased vascular permeability [18–22]. Together, these factors contribute to the progression and worsening of DME in patients with impaired renal function.

Although we only analyzed serum Cre, eGFR as markers of renal function, additional indicators—such as urinary protein and the urinary albumin-to-creatinine ratio (ACR)—are also associated with the risk of developing diabetic macular edema (DME) [3–5, 23, 24]. Further studies are warranted to identify which specific indicators have the most significant impact on the pathogenesis of DME and DR.

### Renal function impairment and responsiveness to anti-VEGF therapy in DME

Our study findings reveal that, although there was no significant association between baseline renal function (serum Cre levels and eGFR) and both visual and morphological outcomes in patients with DME undergoing anti-VEGF treatment, the presence of stage 3 or higher CKD at 36 months was significantly associated with greater fluctuations in CMT.

Although there are several reports on the association between renal function and responsiveness to anti-VEGF therapy for DME, the findings vary, and a consistent consensus has not yet been established, as detailed below. (1) Reports indicating no significant impact of baseline renal function on DME treatment outcomes: A randomized controlled trial of monthly ranibizumab (RISE/RIDE) found that serum Cre and eGFR levels did not affect visual prognosis [12]. (2) Reports indicating a negative impact of decreased renal function on DME treatment outcomes: In a PRN study of conbercept, baseline urinary ACR was positively correlated with CMT and injection frequency [10]. Following three ranibizumab injections, cases with lower eGFR showed a higher presence of subretinal fluid [9]. Post-bevacizumab, reductions in CMT were significantly smaller in cases with higher BUN and Cre values and lower eGFR [25], High baseline urinary ACR cases showed significantly greater CMT fluctuations with PRN therapy (DRCR Protocols T, V) [26]. (3) Reports indicating that decreased renal function positively impacted DME treatment outcomes: In a PRN study with ranibizumab, patients with macroalbuminuria (urinary ACR > 300mg/g) showed significantly greater CMT improvements compared to those with urinary ACR < 30 mg/g [11]. In our study, baseline CKD stage (whether stage 3 or higher or lower) did not significantly affect visual outcomes, maximum CMT during follow-up, or CMT fluctuation at 36 months. However, at 36 months, patients with stage 3 or higher CKD showed significantly greater maximum CMT and fluctuations compared to those with CKD below stage 3.

The finding that baseline renal function did not affect visual prognosis aligns with Singh et al. [12] and our observation of comparable CMT improvements in cases with poor baseline renal function echoes Lai et al*.* [11]. However, our results differ from Liu et al. [10], Tsai et al. [9], Hwang et al. [25], and Torjani et al. [26], who report that poorer baseline renal function was associated with lesser CMT reduction, greater fluctuation, and increased injection frequency with anti-VEGF treatment. Notably, Torjani A et al. and Liu ZY et al. used baseline urinary ACR as an indicator. While urinary ACR was not analyzed in our study, it is an early marker of diabetic nephropathy, indicating early DKD. Our study likely included cases with early DKD at baseline that progressed to stage 3 CKD with decreased eGFR during follow-up. Thus, future investigations incorporating early nephropathy markers like baseline urinary ACR are warranted to track long-term renal function changes.

### Improvement of DME with dialysis

Dialysis initiation for end-stage renal failure is reported to improve DME [27, 28]. The mechanisms underlying the improvement in DME after dialysis initiation include resolution of choroidal edema, improvement of plasma colloid osmotic pressure, resolution of uremic conditions such as hypertension and anemia, and reduction in circulating inflammatory cytokines and growth factors. As previously reported, in our study, all patients who required dialysis during the follow-up period showed a significant decrease in CMT after dialysis initiation, with only one patient requiring additional anti-VEGF injections.

### Renal function deterioration during Anti-VEGF treatment

The annual progression rates from DKD stages 1 to 2, stages 2 to 3, and stages 3 to 4 are reported to be 2%, 2.8%, and 2.3%, respectively, low compared to those in our study [29]. The accelerated progression rate of DKD in this study compared to the general diabetic population can be attributed to two factors: (1) this study focused on patients with DME and DR, both known risk factors for renal function deterioration. Park et al. report that the risk of CKD progression was 2.9 times higher in patients with non-proliferative retinopathy at baseline and 16.6 times higher in patients with proliferative retinopathy at baseline [8]. Additionally, Moriya et al. report that patients with microalbuminuria and retinopathy showed a greater decline in eGFR, suggesting that CKD with DME may progress more rapidly [7].

(2) Considering previous reports on the impact of anti-VEGF agents on renal impairment, it is possible that intravitreal anti-VEGF therapy may have contributed to the worsening of renal function over the course of follow-up. Several reported cases show that some individuals experience decreased eGFR and developed proteinuria after receiving anti-VEGF intravitreal injections, and subsequent renal biopsies revealed glomerular pathological abnormalities such as focal segmental glomerulosclerosis, minimal change disease, and thrombotic microangiopathy [30, 31]. VEGF secreted by podocytes in the glomerulus plays a crucial role in maintaining endothelial cell function. Systemic VEGF inhibition can disrupt podocyte function, leading to a decline in glomerular function [32]. In this study, eight cases progressed to dialysis during follow-up, exhibiting a rapid decline in renal function (Table 5), suggesting that the impact of anti-VEGF therapy on accelerating renal function decline cannot be ruled out. Although there are some case reports on the systemic effects of intravitreal anti-VEGF injections, it remains uncertain whether these effects are directly attributable to the injections.

### Limitations

The retrospective nature of our study introduces potential biases and limitations in the data collection and analysis. Additionally, the relatively small sample size and single-center design may limit the generalizability of our findings. The observational design of this study prevents us from establishing causality between renal function impairment and DME development or worsening. Moreover, the lack of long-term follow-up data limits our understanding of the sustained effects of anti-VEGF therapy and dialysis on DME and renal function. Furthermore, this study was conducted in a real-world setting, where the likelihood of undertreatment was potentially present.

This retrospective observational study provides valuable insights into longitudinal changes in renal function and CMT during anti-VEGF therapy for DME. Our findings highlight the high prevalence of renal function impairment in patients with DME and a marked progression of renal impairment was observed during the course of anti-VEGF therapy. Renal function parameters may influence DME fluctuation. Furthermore, dialysis initiation in patients with end-stage renal failure can lead to an improvement in DME. These findings emphasize the importance of monitoring renal function. Caution should be exercised regarding potential renal complications associated with anti-VEGF intravitreal injections. Further research is required to elucidate the complex relationship between renal function, DME, and the efficacy of anti-VEGF therapy.
